# Essentiality of *mmpL3* and impact of its silencing on *Mycobacterium tuberculosis* gene expression

**DOI:** 10.1038/srep43495

**Published:** 2017-02-27

**Authors:** Giulia Degiacomi, Andrej Benjak, Jan Madacki, Francesca Boldrin, Roberta Provvedi, Giorgio Palù, Jana Kordulakova, Stewart T. Cole, Riccardo Manganelli

**Affiliations:** 1 Department of Molecular Medicine, University of Padova, Padova, Italy; 2 Global Health Institute, Ecole Polytechnique Fédérale de Lausanne, Lausanne, Switzerland; 3 Department of Biochemistry, Faculty of Natural Sciences, Comenius University in Bratislava, Bratislava, Slovakia; 4 Department of Biology of Padova, Padova, Italy

## Abstract

MmpL3 is an inner membrane transporter of *Mycobacterium tuberculosis* responsible for the export of trehalose momomycolate, a precursor of the mycobacterial outer membrane component trehalose dimycolate (TDM), as well as mycolic acids bound to arabinogalactan. MmpL3 represents an emerging target for tuberculosis therapy. In this paper, we describe the construction and characterization of an *mmpL3* knockdown strain of *M. tuberculosis*. Downregulation of *mmpL3* led to a stop in bacterial division and rapid cell death, preceded by the accumulation of TDM precursors. MmpL3 was also shown to be essential for growth in monocyte-derived human macrophages. Using RNA-seq we also found that MmpL3 depletion caused up-regulation of 47 genes and down-regulation of 23 genes (at least 3-fold change and false discovery rate ≤1%). Several genes related to osmoprotection and metal homeostasis were induced, while several genes related to energy production and mycolic acids biosynthesis were repressed suggesting that inability to synthesize a correct outer membrane leads to changes in cellular permeability and a metabolic downshift.

Tuberculosis (TB) is the leading cause of death worldwide due to an infectious disease, surpassing malaria and HIV, with an estimated 10.4 million new cases of active TB and 1.8 million deaths in 2015^1^,^2^. WHO estimates that there were 480,000 new cases of multidrug-resistant (MDR) tuberculosis and an additional 100,000 people with rifampicin-resistant TB^1^. Lately, considerable progress was achieved: several new or repurposed antimicrobial drugs are in advanced stages of clinical trials for MDR-tuberculosis, and two new antimicrobial drug candidates are in late-stage trials^2^. However, this is not sufficient to reach the goal of the eradication of TB by 2030. The low treatment success rates for MDR- and XDR-tuberculosis as well as HIV co-infections illustrate the urgent need for development of new anti-tubercular drugs, adjunct therapies, and vaccines to improve treatment outcomes.

The discovery and characterization of novel targets is a fundamental step for the development of drugs with new mechanisms of action and thus without cross-resistance problems. Recently, MmpL3, a transporter from the Mycobacterial Membrane Protein Large (MmpL) family, specific for Actinobacteria, was identified in high-throughput whole-cell screens as the target of several potent anti-mycobacterial agents^3^,^4^,^5^,^6^,^7^,^8^,^9^,^10^,^11^. MmpL proteins belong to the resistance-nodulation-cell division (RND) permease superfamily and are important in substrate transport across the inner membrane. Of the 13 MmpL proteins encoded in the *Mycobacterium tuberculosis* genome only MmpL3, shown to export trehalose monomycolate (TMM), the precursor of trehalose dimycolate (TDM) and mycolates bound to arabinogalactan^3^,^12^, was suggested to be essential^13^. The recently identified MmpL3 inhibitors are present as a variety of chemical scaffolds and differ in their spectrum of activity, raising the question whether they exert their action through specific inhibition of MmpL3 transporter functions or through an indirect mechanism involving the dissipation of the proton motive force^14^,^15^. MmpL3 is also an important drug target in non-tuberculous mycobacteria such as *Mycobacterium abscessus* for which treatment options are severely limited^16^.

The essentiality of *mmpL3* for growth in axenic media and for establishing infection in a mouse model was recently demonstrated using a conditional knockdown mutant^17^. Moreover, it was also shown that depletion of MmpL3 had a rapid bactericidal effect and further rendered *M. tuberculosis* hyper-susceptible to MmpL3 inhibitors, underlining the therapeutic potential of MmpL3^17^. In this report, we constructed and characterized a similar conditional mutant confirming the essentiality of MmpL3 for growth in axenic media and its role in TMM export, and also demonstrate its essentiality for intracellular growth. Finally, we applied a transcriptomic approach to better characterize its physiological role under standard laboratory growth conditions.

## Experimental Procedures

### Bacterial strains, media and growth conditions

*M. tuberculosis* H37Rv and its derivative strains were routinely cultured at 37 °C in either Middlebrook 7H9 (liquid medium) or 7H10 (solid medium, Difco), supplemented with 0.05% v/v Tween 80 (Sigma-Aldrich), 0.2% v/v glycerol (Sigma-Aldrich) and 10% ADN (2% glucose, 5% BSA, 0,85% NaCl).

For cloning procedures, *Escherichia coli* DH5α was grown in Luria Bertani (LB) broth and LB-agar. When required, antibiotics were added at the following concentrations: kanamycin (Sigma-Aldrich), 50 μg/ml; hygromycin (Invitrogen), 200 μg/ml (*E. coli*); streptomycin (Sigma-Aldrich), 50 μg/ml (*E. coli*); streptomycin, 20 μg/ml (*M. tuberculosis*); hygromycin, 50 μg/ml (*M. tuberculosis*). Anhydrotetracycline (ATc, Sigma) was added, when required, at a final concentration of 500 ng/ml.

### *M. tuberculosis mmpL3* conditional mutant construction

The *mmpL3* conditional mutant was constructed using the TetR/Pip OFF repressible promoter system^18^. For this purpose, the first 787 bp of *mmpL3* were amplified using primers: RP1576: 5′-TTTTATGCATTTCGCCTGGTGGGGTCGAACTG-3′ and RP1577 5′-ACTAGTCTCTTCGCGGAACCGGCTCA-3′, and cloned downstream of the repressible promoter P_*ptr*_ in a suicide plasmid conferring resistance to hygromycin. Ten μg of the resulting plasmid were introduced by electroporation into TB38, an *M. tuberculosis* strain with the TetR/Pip OFF system integrated into its genome and carrying a streptomycin resistant determinant^18^. Transformants were selected on 7H10 agar plates containing streptomycin and hygromycin and were screened by PCR, using an upper primer in the TetR-P_*ptr*_ region (5′-CCTGACGGATGGCCTTACGAGTT-3′, RP1666) and a lower primer external to the homology region used for recombination (5′-AGACAGGATGGCCGACAGCAT, RP1575) (data not shown). One representative mutant in which the *mmpL3* physiologic promoter was replaced by P_*ptr*_ by insertional duplication was selected and named TB416.

### RNA extraction

Exponential phase *M. tuberculosis* cultures were pelleted and cells were flash frozen in liquid nitrogen and stored at −80 °C until use. Bacteria were re-suspended in 1 ml Trizol (Ambion) and added to a 2-ml screw-cap tube containing 0.5 ml zirconia beads (BioSpec Products) for disruption by bead-beating (twice for 1 minute with a 2-minute interval on ice). The cell suspension was then transferred to a new tube, where chloroform-isoamylalcohol (24:1) extraction was performed. RNA was precipitated by adding 1/10 volume of sodium acetate (2 M, pH 5.2) and 0.7 volume of isopropanol, washed with 70% ethanol, air-dried and resuspended in DEPC-treated water. DNase treatment was carried out using TURBO DNA-free™ Kit (Ambion), following the manufacturer’s recommendations, and the reactions were subsequently cleaned up by phenol-chloroform extraction and ethanol precipitation. RNA was stored at −80 °C in DEPC-treated water. Amount and purity of RNA were determined spectrophotometrically; integrity of RNA was assessed on a 1% agarose gel.

### Library preparation for RNA-seq analysis and RNA-seq data analysis

The *mmpL3* conditional mutant TB416 was grown until exponential phase with or without ATc [500 ng/ml] in roller bottles (two biological replicates per condition). As a control for the effect of ATc alone, ATc-treated and untreated parental strain was used (one sample per condition). Total RNA was extracted as previously described, and used for the preparation of strand-specific Illumina libraries, with an additional ribosomal RNA depletion step. Ribosomal transcript depletion was performed on 1 μg of total RNA using the Ribo-Zero rRNA Removal Kit for Gram-Positive Bacteria (Catalog Number MRZGP126; Illumina, San Diego, USA) according to the protocol provided by the supplier. Sequencing libraries were then generated using the resulting ribosomal transcript-depleted RNA and the Illumina TruSeq Stranded mRNA Library Prep Kit reagents (Catalog Number RS-122-2101) according to the protocol provided by the supplier. Sequencing cluster generation was performed with the resulting libraries and the Illumina TruSeq SR Cluster Kit v4 reagents (Catalog Number GD-401-4001) and sequenced on the Illumina HiSeq 2500 using TruSeq SBS Kit V4 reagents (Catalog Number FC-401-4002) as single-end 100 nt-long reads. Sequencing data were processed using the Illumina Pipeline Software version 1.84. Reads were right-trimmed for the Illumina adapter sequence using Flexbar (https://sourceforge.net/projects/flexbar/) and aligned with Bowtie2^19^. Counting reads over annotated features was done with featureCounts^20^. Annotation was taken from TubercuList release R27. To avoid the influence of transcripts deriving from the original 5′ fragment of *mmpL3*, a truncated version of *mmpL3* was added to the annotation. Differential gene expression (DGE) analysis was done using DESeq2^21^. Raw and processed data were deposited in the GEO database (https://www.ncbi.nlm.nih.gov/geo/)^22^ as Series record GSE89830.

### Quantitative reverse transcription-PCR (qRT-PCR)

qRT-PCR was performed as previously described using the primers shown in Supplementary Table S1. *sigA* mRNA was used as an internal invariant control for data normalization^23^. RNA samples that had not been reverse transcribed were included in all experiments to exclude significant DNA contamination. For each sample, melting curves were performed to confirm the purity of the products.

### Analysis of the lipid composition of the *mmpL3* conditional knockdown strain TB416

TB416 was grown at 37 °C with shaking in Middlebrook 7H9 with/without ATc [500 ng/ml]. After 96 hours, cells were harvested by centrifugation and pellets used for analysis. Cultures of *M. tuberculosis* H37Rv were grown in the same conditions and used as a control. The cells were delipidated by 2 hrs extractions with 6 ml of chloroform/methanol (1/2) and subsequently 2 × 6 ml of chloroform/methanol (2/1) and stirring at 56 °C. Extracted lipids were subjected to biphasic washes using chloroform/methanol/water (4:2:1)^24^, dried under N_2_ and resolved in chloroform/methanol (2/1); 100 μl of solvent was used to extract material from cells grown in 30 ml culture, OD_540_ = 0.64. Five μl of each sample were loaded on a TLC plate, which was developed in chloroform/methanol/water (20/4/0.5). Lipids were visualized by spraying with CuSO_4_ (10% in 8% phosphoric acid solution) and heating.

Moreover, pellets from the same cultures were used to analyse cell wall-bound mycolic acids, as well as mycolic acids in the form of extractable lipids. For this aim, delipidated cell pellets and 50% of extracted lipids were subjected to alkali saponification in 2 ml of 15% TBAH at 100 °C overnight. Mycolic acid methyl esters (MAMEs) were prepared according to Phetsuksiri and collaborators^25^. Briefly, after cooling, 3 ml of dichloromethane, 2 ml of water and 300 μl of iodomethane were added to each sample and the mixtures were shaken by rotating for 4 hours at RT. The samples were centrifuged, the upper water phases were removed and organic phases were twice washed by water. Lower organic phases were dried under N_2_ and MAMEs were extracted by adding 3 ml of diethylether and sonication. After centrifugation, the extracts were removed, dried under N_2_ and resolved according to the OD_540_ of harvested cultures as described above. 5 μl of cell wall-bound MAMEs and 10 μl of methylesters from extractable lipids were loaded on a TLC plate, developed in n-hexane/ethyl acetate (95/5, 3x) and visualized with CuSO_4_.

### Infection of macrophages

THP-1 monocytes (American Type Culture Collection) were grown in suspension at 37 °C in 5% CO_2_ in bicarbonate-buffered RPMI (Gibco), supplemented with 10% (vol/vol) fetal bovine serum (FBS) (Gibco), 50 μM β-mercaptoethanol, up to a density of about 0.5 × 10^6^ cells/ml. Differentiation of monocytes into macrophages was achieved by plating the cells in 96-well plates at a density of 7.5 × 10^4^ cells/well in the presence of 50 ng/ml phorbol1 2-myristate 13-acetate (PMA) (Sigma-Aldrich). After 24 hours, PMA was removed and cells were infected with *M. tuberculosis* strains at a multiplicity of infection of 1:20 (CFU:cell) for 90 min as previously described^26^. After infection, extracellular bacteria were removed by washing twice with PBS, then fresh medium with or without 200 ng/ml ATc was added. The medium was replaced every 48 hours. At different time points macrophages were lysed and serial dilutions of extracts plated on 7H10 medium to determine viable counts.

## Results and Discussion

### Generation and characterization *in vitro* of *mmpL3* conditional mutant in *M. tuberculosis*

**Figure 1:**
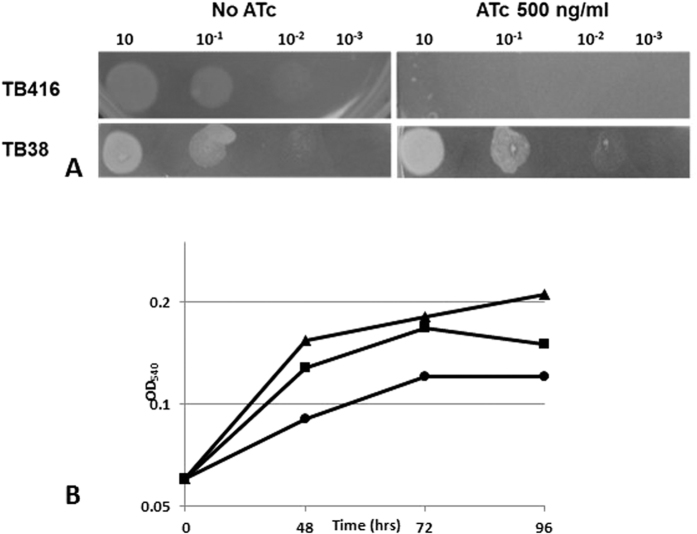
(**A**) Serial dilutions of log-phase cultures of the *mmpL3* conditional mutant TB416 and its parental strain TB38 strains were spotted on Middlebrook 7H10 plates with or without 500 ng/ml ATc; (**B**) Growth curves of standing cultures of the *mmpL3* conditional mutant in the presence of different ATc concentrations. Circles: ATc 500 ng/ml; squares: ATc 100 ng/ml; triangles: no ATc. The experiment was repeated three times giving comparable results. The figure shows one representative experiment.

**Figure 2:**
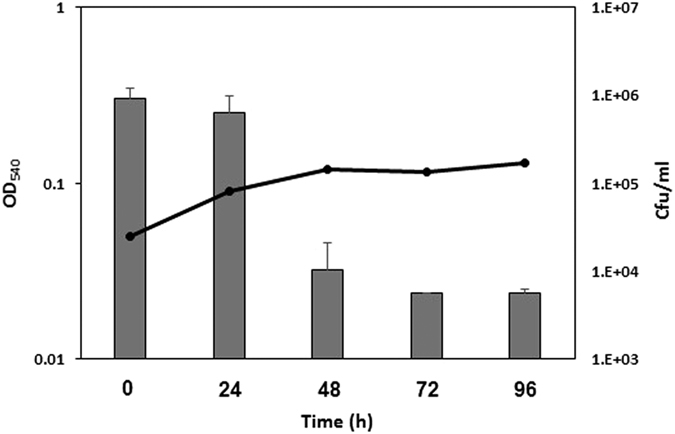
Killing of the *mmpL3* conditional mutant in the presence of ATc. The mutant was grown in roller bottles in Middlebrook 7H9 containing 500 ng/ml ATc. When the culture reached mid-log phase, bacteria were diluted in fresh media containing ATc (T0). Samples were collected at different time points and plated on Middlebrook 7H10 to determine the number of colony forming units (CFU). The line represents the optical density of the culture, while bars represent the CFU/ml. The experiment was repeated four times and comparable results were obtained. The figure shows one representative experiment.

A repressible *mmpL3* conditional knockdown (cKD) mutant (TB416) was constructed using the TetR/Pip OFF repressible promoter system, allowing target gene repression in the presence of ATc^18^. Unlike its parental strain, the conditional mutant TB416 was not able to grow on plates containing ATc (Fig. 1A). Accordingly, its growth was also inhibited in a dose-dependent manner in the presence of ATc in liquid media (Fig. 1B). We also analyzed the cell viability upon ATc addition, as reported by the number of colony-forming units (cfu), and confirmed that repression of *mmpL3* leads to bacterial death, with a drop in viability of about 160-fold after 72 hours of exposure to ATc (Fig. 2). It should be noted that while the number of cfu dropped dramatically, the optical density remained stable, suggesting that MmpL3 depletion did not kill the cells through cell lysis. The number of cfu surviving *mmpL3* depletion levels off between 72 and 96 h of ATc exposure suggesting the presence of a bacterial subpopulation able to survive (even if they cannot grow) in the presence of only residual activity of *mmpL3* expression.

### Analysis of the lipid composition of the *mmpL3* cKD strain

**Figure 3:**
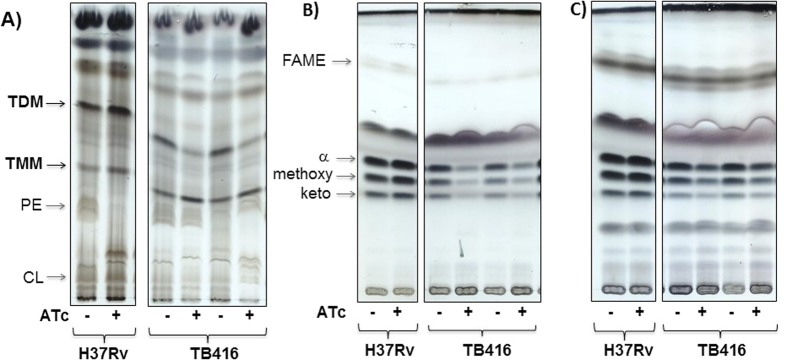
Accumulation of TMMs in the MmpL3-depleted strain. (**A**) Lipids were extracted from *M. tuberculosis* TB416, grown in the absence and presence of ATc (ATc−/+), with CHCl_3_/CH_3_OH (1:2–1x; 2:1–2x), then separated by TLC in the solvent CHCl_3_/CH_3_OH/H_2_O (20:4:0.5) and detected with 10% (w/v) CuSO4 in 8% (v/v) H_3_PO_4_ for the complete lipid profiles. From the same cultures, delipidated cells, as well as lipid fractions were saponified with 15% TBAH. Free fatty/mycolic acids were derivatized to methylesters, analysed by TLC in n-hexane:ethyl acetate (95:5, 3x) and detected with a CuSO_4_ detection system. (**B**) Mycolic acids bound to cell wall. (**C**) Fatty and mycolic acids from extractable lipids. Samples are in duplicates. H37Rv was used as a control. TDM: trehalose dimycolate; TMM: trehalose monomycolate; PE: phosphatidylethanolamine; CL: cardiolipin; FAME: fatty acid methyl ester; α, methoxy, keto: respectively alpha-, methoxy- and keto-mycolic acids (MAME, mycolic acid methyl ester).

We analysed both the global lipid profile and the mycolic acids content of *mmpL3* cKD strain upon ATc addition. The lipids extracted from cultures grown with or without ATc for 96 hours were examined by TLC. Consistent with the results published by Li and collaborators^17^, the *mmpL3* cKD strain grown in the presence of ATc showed significant changes in the composition of the mycobacterial cell envelope, clearly producing less TDM and accumulating TMM in comparison with the untreated control and with the wild-type strain, as shown in Fig. 3. Moreover, we also observed significantly decreased amounts of cell wall bound mycolates in the *mmpL3* cKD strain grown in the presence of ATc compared to the control strains. These observations confirmed that depletion of MmpL3 leads to abolished flipping of TMM through the membrane resulting in a shortage of mycolic acids bound to arabinogalactan, as well as in the form of TDM. In panel A an unknown lipid running between phosphatidyl-ethanolamine and cardiolipin accumulates in H37Rv grown in the presence of ATc and in the mutant grown in the absence of ATc. The presence of this band varied between different samples (data not shown) and was neither associated with the presence of ATc nor MmpL3 depletion, so its nature was not further investigated.

### Survival of *M. tuberculosis* in THP-1 derived macrophages upon MmpL3 depletion

**Figure 4:**
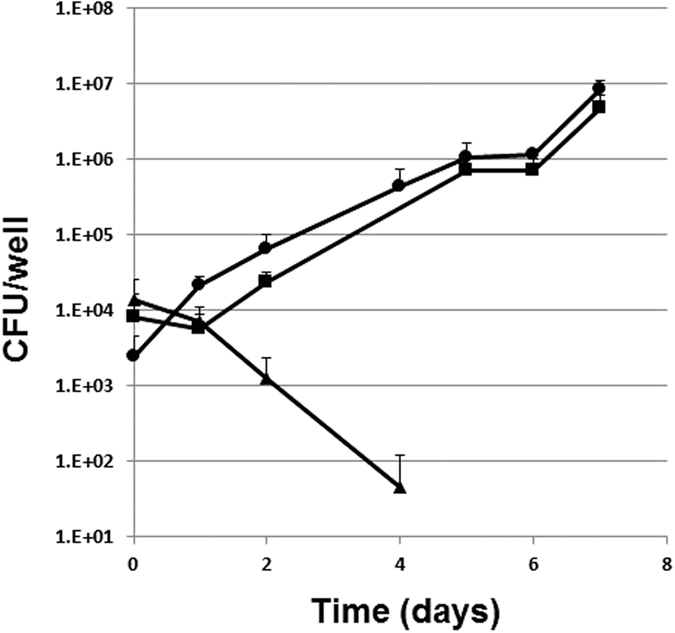
Growth of the *mmpL3* cKD mutant and its parental strain TB38 in THP-1-derived macrophages. Infection was performed at an MOI of 1:20 (bacteria/macrophage). The results are expressed as CFU/well. ATc (200 ng/ml) was added to the cell culture medium, when appropriate. Two separate infections were performed in duplicate. Triangles: *mmpL3* mutant plus ATc; circles: parental strain TB38 plus ATc; squares: *mmpL3* mutant with no ATc. While colonies were detectable until 4 days after infection starting from day 5 no colonies were detected from cell cultures infected with the mutant strain and treated with ATc (detection limit 20 cfu/well).

To assess the essentiality of MmpL3 during intracellular growth, THP-1-derived human macrophages were infected with the *mmpL3* cKD mutant and its parental strain at a multiplicity of infection (MOI) of 1:20 (bacteria per cell) and their growth was followed for a week in the presence or absence of ATc [200 ng/ml] in the cell culture medium. As shown in Fig. 4, the *mmpL3* cKD was able to grow intracellularly to the same level as its parental strain. However, when ATc was added to the cell culture the number of viable counts of the cKD mutant dropped more than 100-fold during the first 4 days to below the limit of detection (20 cfu/well) from day 5 until the end of the experiment, thereby showing the essentiality of MmpL3 for intracellular growth and survival.

### Transcriptomic response to *mmpL3* depletion

The characterization of the global transcriptional response to MmpL3 depletion can lead to better knowledge of the physiological functions of MmpL3 and give hints on the physiological state of cells treated with drugs able to inhibit MmpL3 functions. Total RNA was extracted and analyzed by RNA-seq from TB416 cultures grown to mid-exponential phase (OD_540_ 0.3) in the presence or absence of ATc 500 ng/ml. Cells were collected before MmpL3 depletion caused growth arrest and bacterial death. The complete set of data is shown in Supplementary dataset 1. We could not detect any significant variation of gene expression levels in TB416 parental strain in response to ATc. However, treatment of the conditional mutant TB416 with ATc caused up-regulation of 47 genes and down-regulation of 23 genes (at least 3-fold change and false discovery rate ≤1%), listed in Supplementary dataset 2 and 3, respectively. Five differentially expressed genes (two induced and three repressed) were selected and their level of expression was analyzed by quantitative real-time PCR (qRT-PCR), confirming the RNA-seq data (Supplementary Table S2).

### Induced genes

Out of eleven functional categories defined for *M. tuberculosis*^27^, one category: “regulatory proteins” was significantly enriched (“N-1” Chi-squared test, P = 0.0005) in the 47 up-regulated genes (17.02%) compared to its representation in the genome (4.82%). Twenty-six genes were located in nine clusters of adjacent genes suggesting their co-transcription. The most up-regulated gene was *rv1057* (11.8 fold), coding for a protein of unknown function with a β-propeller structure. This gene was previously found to be induced in *M. tuberculosis* strains 18b and H37Rv under conditions of surface stress and was hypothesized to be under the transcriptional regulation of MprA and SigE^26^,^28^,^29^.

Interestingly, several genes and operons up regulated in response to MmpL3 depletion are known to be induced by copper, as the *cso* operon, encoding a copper export system, *cadI* and *arsC* (encoding putative metal transporters), *rv2963* (coding for a putative ABC permease), *mymT* (encoding a metallothionein protecting cells from copper toxicity), *mmcO* (encoding a multicopper oxidase required for copper resistance)^30^ and the adjacent, but divergently transcribed operon *lpqS-cysK2-rv0849-rv0850*, essential for virulence and copper resistance^31^,^32^,^33^. Interestingly, the latter operon has also been shown to be induced in response to hypoxia and oxidative stress, and contains a gene, *cysK2,* whose product is involved in the biosynthesis of S-sulfocysteine, a precursor of mycothiol that has been suggested to act as a signaling molecule triggering additional responses upon exposure to reactive oxygen species during dormancy^34^. Taken together, these data suggest that MmpL3 depletion affects cell permeability and perturbs metal homeostasis and, consequently, the cytoplasmic redox potential.

Moreover, another induced gene was *rv0516c*, encoding a SigF anti-anti–σ-factor. Rv0516c was recently renamed osmosensory protein A (OprA) for its involvement in an osmosensory signaling pathway including PknD and SigF, which regulates peptidoglycan architecture^35^, suggesting that cells depleted in MmpL3 might experience osmotic stress, probably due to a change in surface permeability as a result of a different lipid composition. Consistent with this hypothesis, *argC,* the first gene of the arginine biosynthetic operon was found to be induced 3.9-fold: L-arginine is considered to be an osmoprotectant and induction of its biosynthetic operon was reported under conditions of osmotic stress^35^. Another sign of surface stress was the induction of the *iniBAC* operon, defined as a general cell wall stress operon that responds to cell wall biosynthesis inhibition upon addition of EMB and INH^36^,^37^ and recently shown to respond to mutations that cause defects in the biosynthesis of methyl-branched lipids^38^.

Upregulated genes also included several transcriptional regulators; particularly interesting was the induction of the genes encoding WhiB6 and WhiB7. The former has been recently shown to regulate the ESX-1 and DosR regulons in response to nitric oxide thereby modulating granuloma formation and virulence^39^, while the latter is known to regulate several drug resistance genes^40^, suggesting that MmpL3 depletion might interfere with virulence and with drug sensitivity. Finally, among the up-regulated mRNA we also found a small RNA: ASdes (MTB000053), which was previously shown to be an antisense RNA to *desA1*, encoding an acyl-carrier protein desaturase involved in mycolic acid biosynthesis, and with significant complementarity also to *desA2*, encoding a second acyl-carrier protein desaturase, thus suggesting a role in regulation of lipid metabolism^41^,^42^. Interestingly, both *desA1* and *desA2* were down-regulated (2.7 and 2.6-fold, respectively) after *mmpL3* depletion (Supplementary dataset 1).

### Repressed genes

The most represented functional category among genes repressed at least 3-fold was that of “lipid metabolism” (8 genes = 34.78%), which was significantly enriched compared to its representation in the genome (6.61%, P < 0.0001 in “N-1” Chi-squared test). Fifteen genes were located in 6 clusters of adjacent genes that are likely to be co-transcribed (Supplementary dataset 3). As expected, one of the most repressed genes was indeed *mmpL3*. In accordance with MmpL3 function, several genes encoding proteins involved in mycolic acid and TDM biosynthesis were repressed (*e.g. acpM-kasA-kasB,* or *fbpB*) suggesting the presence of feedback mechanisms downregulating this biosynthetic pathway as a consequence of the accumulation of TMM in the cytoplasm. Several other repressed genes, however, were involved in energy production such as *sdaA*, involved in gluconeogenesis from serine^43^, *atpB,* encoding a component of ATP synthase, *nuoB, D* and *H* encoding different subunits of NADH dehydrogenase, or the entire *mce1* operon, hypothesized to encode a lipid re-uptake system that enables mycolic acid recycling^44^, suggesting a decrease in the metabolic activity of the cells.

## Conclusions

After constructing an *M. tuberculosis mmpL3* cKD mutant, we confirmed that MmpL3 is essential for growth in axenic culture and for transport of TMM^17^. We proved its essentiality for growth and survival of *M. tuberculosis* during human macrophage infection and showed that MmpL3 depletion leads to a complex change in the global transcriptional profile. In agreement with MmpL3′s role in TMM export and its essentiality for cell replication, its depletion led to the repression of genes involved in mycolic acid and TDM biosynthesis as well as down-regulation of genes involved in energy production and respiration, probably in an attempt to reduce growth rate and oxidative stress in response to defective export of essential cell wall components. Analysis of the induced genes, however, suggested that MmpL3 depletion led to a modification of surface permeability, probably due to the inability to construct the cell wall correctly, leading to osmotic stress and disruption of metal homeostasis. These data suggest that the change in permeability due to the impossibility to build a correct cell wall is the main mechanism by which MmpL3 depletion affects cellular viability. Taken together, our findings, and those of others^17^, underline the vulnerability of MmpL3 and the ensuing implications for drug development for tuberculosis and infections due to difficult to treat pathogens such as *M. abscessus*.

## Additional Information

**How to cite this article:** Degiacomi, G. *et al*. Essentiality of *mmpL3* and impact of its silencing on *Mycobacterium tuberculosis* gene expression. *Sci. Rep.* **7**, 43495; doi: 10.1038/srep43495 (2017).

**Publisher's note:** Springer Nature remains neutral with regard to jurisdictional claims in published maps and institutional affiliations.

## Supplementary Material

Supplementary Tables

Supplementary Dataset 1

Supplementary Dataset 2

Supplementary Dataset 3
