# Supplementary material for: Essentiality of mmpL3 and impact of its silencing on Mycobacterium tuberculosis gene expression
Source: Sci Rep. 2017 Feb 27;7:43495. doi: 10.1038/srep43495 (PMC5327466; doi:10.1038/srep43495)
Supplement: Supplementary Tables [file srep43495-s1.pdf]

# Essentiality of *mmpL3* and impact of its silencing on *Mycobacterium tuberculosis* gene expression

**Giulia Degiacomi[1], Andrej Benjak[2], Jan Madacki[3], Francesca Boldrin[1], Roberta Provvedi[4], Giorgio Palù[1], Jana Kordulakova[3], Stewart T. Cole[2], Riccardo Manganelli*[1]**

[1]Department of Molecular Medicine, University of Padova, Padova, Italy; [2]Global Health Institute, Ecole Polytechnique Fédérale de Lausanne, Lausanne, Switzerland; [3]Department of Biochemistry, Faculty of Natural Sciences, Comenius University in Bratislava, Bratislava, Slovakia; [4]Department of Biology of Padova, Padova, Italy.

**Table S1. Oligonucleotides used for qRT-PCR**

| Name | Sequence 5'→3' | Target gene |
|---|---|---|
| RP1843 | cgggatcagcgagaagtact | *mmpL3* |
| RP1844 | gttgtccggctcgatgaatc | |
| RP1516 | ccatcccgaaaaggaagacc | *sigA* |
| RP1517 | aggtctggttcagcgtcgag | |
| RP1825 | tcgttcggatgctggagtc | *csoR* |
| RP1826 | caatgagctcttcgatggcc | |
| RP1827 | gctcaaacagggcaagagtc | *iniA* |
| RP1828 | catcggtgctgatgtcatcg | |
| RP1833 | ttcgggatggaacggttctc | *nuoB* |
| RP1834 | agatgtcgaccggaacaaca | |
| RP1835 | gcggacatcaattacgtgct | *atpB* |
| RP1836 | cgatcagtgcgaccagaatg | |
| RP1837 | caagtgggatctagcggtca | *kasA* |
| RP1838 | cgcattcatcaggtcgtagc | |

| **Table S2. Validation of RNA-seq data with qRT-PCR** | | | |
|---|---|---|---|
| **Gene** | **Gene name** | **FoldChange Rna-seq** | **FoldChange qRT-PCR** |
| Rv0967 | *csoR* | 7.12 | 3.37 ± 0.90 |
| Rv0342 | *iniA* | 5.38 | 9.2 ± 1.54 |
| Rv3146 | *nuoB* | 0.29 | 0.51 ± 0.25 |
| Rv1304 | *atpB* | 0.32 | 0.21 ± 0.02 |
| Rv2245 | *kasA* | 0.33 | 0.53 ± 0.2 |
